# Correspondence

**Published:** 1868-09

**Authors:** Charles A. Lee


### Correspondence.

To the Buffalo Medical and Surgical Journal:

Mr. Editor:—You quote from a recent number of the "*Nashville Republican Banner*," a notice of my Address before the "American Teachers' Association," at its late annual meeting, wherein it is stated that I "severely criticised the pernicious practice of students in schools and colleges, spending their time in idle lassitude, and indulging in the deleterious habits of chewing and smoking tobacco, and in other *no less wicked amusements.*"

Now, though the matter is not of much consequence, I would not like to be misrepresented in your columns, as I certainly am in this extract. My remarks were wholly devoted to the subject of *School-Hygiene*, and the great neglect of teachers in regard to sanitary arrangements of schools, etc. I tried to show that there were few schools, from the humblest primary school to the college and university, where a thoroughly enlightened regard for the physical welfare of the student was manifested. That either the school-site was unsuitable, or the school-rooms badly ventilated, lighted and warmed; or the construction of the desks and seats faulty; or the hours of study excessive; or, if a boarding-school, the diet was insufficient and improper; or, the character and amount of exercise not well regulated; or, the attention to personal cleanliness neglected; or the building improperly planned and unsuitable; or, injurious punishments inflicted; that in these and many other respects, which I pointed out, the health of pupils suffered, and often the foundation of disease was laid, from which they never recovered. I insisted, particularly, on the fact, that more time is given to study in our schools, and *out of it*, in consequence of lessons being given out to be learned at home, than was consistent with health and permanent intellectual progress. I referred to the normal schools of Massachusetts and New England, particularly, in illustration, and quoted the last Report of the "*Massachusetts Board of Education,*" to substantiate my statements. I endeavored to show the ill-effects of confinement and hard study in the New England schools, and that this was probably one of the most prevalent predisposing causes of insanity.

On this subject, as near as I can recollect, I remarked that however much physicians might differ on other subjects, they all agreed as to the destructive effects of premature or excessive mental effort; that though study and bad confinement in schools, was not often one of the immediate or exciting causes of insanity, yet that next to intemperance and hereditary tendency, it was one of the most frequent and influential; moreover, it diminished the conservative power of the animal economy to that degree, that disease, which otherwise would have passed off safely, often destroyed life before danger was anticipated. I stated that insanity was increasing at a fearful rate in New England, so that in Massachusetts and Connecticut, more than 1 in 500 of the population was afflicted with the loss of reason, whereas in the middle, western and southern States it was in a ratio of 1 to 1,000 or 1,500, and it was worth our serious inquiry, whether the principal cause *might not be found connected with the faulty management and education of children.*

Such were the principal topics of my discourse, which was listened to very attentively by the large and intelligent audience, and which I hope was productive of some good, as it led to a very animated discussion of the various matters touched upon, which was again renewed at a subsequent meeting. Some of the teachers from New England, especially Mr. Hagar, the intelligent principal of the "Massachusetts Normal School," in Boston, thought I bore down rather hard upon the normal schools of that State, but as I had merely quoted from the last Report of the "Massachusetts Board of Education," all the statements I had made on the subject, I only had to shift my responsibility on to the respectable gentlemen who drew up said Report, to battle it at their leisure.

As to the habit of *tobacco chewing and smoking*, which your readers might infer, constituted the chief topic of my discourse, I only mentioned the word *tobacco* once, and then very incidentally. If I supposed my remarks had any special interest to medical men, I would quote at greater length. The above, however, may suffice to place me *rectus in curia*, as we say.

CHARLES A. LEE, M. D.

